# The central role of the glutamate metabolism in long-term antiretroviral treated HIV-infected individuals with metabolic syndrome

**DOI:** 10.18632/aging.203622

**Published:** 2021-10-11

**Authors:** Marco Gelpi, Flora Mikaeloff, Andreas D. Knudsen, Rui Benfeitas, Shuba Krishnan, Sara Svenssson Akusjärvi, Julie Høgh, Daniel D. Murray, Henrik Ullum, Ujjwal Neogi, Susanne D. Nielsen

**Affiliations:** 1 Department of Infectious Diseases, Copenhagen University Hospital Rigshospitalet, Copenhagen, Denmark; 2 The Systems Virology Laboratory, Division of Clinical Microbiology, Department of Laboratory Medicine, Karolinska Institute, ANA Futura, Campus Flemingsberg, Stockholm, Sweden; 3 National Bioinformatics Infrastructure Sweden (NBIS), Science for Life Laboratory, Department of Biochemistry and Biophysics, Stockholm University, Stockholm S-10691, Sweden; 4 Centre for Health and Infectious Diseases Research (CHIP), Rigshospitalet, Copenhagen DK-2100, Denmark; 5 Department of Clinical Immunology, Copenhagen University Hospital, Copenhagen, Denmark; 6 Christopher S. Bond Life Sciences Center, University of Missouri, Columbia, MO 65211, USA

**Keywords:** antiretroviral therapy, HIV-infection, metabolic syndrome, metabolomics, immune-phenotyping

## Abstract

Metabolic syndrome (MetS) is a significant factor for cardiometabolic comorbidities in people living with HIV (PLWH) and a barrier to healthy aging. The long-term consequences of HIV-infection and combination antiretroviral therapy (cART) in metabolic reprogramming are unknown. In this study, we investigated metabolic alterations in well-treated PLWH with MetS to identify potential mechanisms behind the MetS phenotype using advanced statistical and machine learning algorithms.

We included 200 PLWH from the Copenhagen Comorbidity in HIV-infection (COCOMO) study. PLWH were grouped into PLWH with MetS (*n* = 100) defined according to the International Diabetes Federation (IDF) consensus worldwide definition of the MetS or without MetS (*n* = 100). The untargeted plasma metabolomics was performed using ultra-high-performance liquid chromatography/mass spectrometry (UHPLC/MS/MS) and immune-phenotyping of Glut1 (glucose transporter), xCT (glutamate/cysteine transporter) and MCT1 (pyruvate/lactate transporter) by flow cytometry. We applied several conventional approaches, machine learning algorithms, and linear classification models to identify the biologically relevant metabolites associated with MetS in PLWH.

Of the 877 identified biochemicals, 9% (76/877) differed significantly between PLWH with and without MetS (false discovery rate < 0.05). The majority belonged to amino acid metabolism (43%). A consensus identification by combining supervised and unsupervised methods indicated 11 biomarkers of MetS phenotype in PLWH. A weighted co-expression network identified seven communities of positively intercorrelated metabolites. A single community contained six of the potential biomarkers mainly related to glutamate metabolism. Transporter expression identified altered xCT and MCT in both lymphocytic and monocytic cells. Combining metabolomics and immune-phenotyping indicated altered glutamate metabolism associated with MetS in PLWH, which has clinical significance.

## INTRODUCTION

With the introduction of combination antiretroviral treatment (cART), people living with HIV (PLWH) have experienced a dramatic increase in life expectancy, with a concomitant decline in AIDS-defining morbidity and mortality [1]. A simultaneous rise in non-AIDS-associated comorbidities has been described, with a particular increment in cardiometabolic diseases, which is now one of the barriers to healthy aging and leading causes of death in well-treated PLWH [2, 3]. The increased risk of metabolic syndrome (MetS) associated with HIV infection [4] is well documented and adversely affects the cardiovascular risk profile. However, there are still numerous gaps in understanding the pathogenesis of MetS in PLWH. Both HIV-specific (cART, ongoing HIV replication, altered gut microbiota, and immunodeficiency) and non-HIV-specific factors (e.g., lifestyle) [4, 5] have been suggested to be involved in the development of MetS in the context of HIV infection, but its exact pathogenesis remains elusive.

With the advancement of technology, high-throughput untargeted metabolomics became attractive as a novel tool to identify large numbers of metabolites and investigate molecular mechanisms of disease phenotypes that are not usually included in routine biochemical analyses [6]. The use of global untargeted metabolomics investigation has a central role in identifying novel biomarkers and potential therapeutic targets in different conditions, including type 2 diabetes and obesity [7–9]. However, no studies are available investigating alterations in the metabolome associated with MetS in the context of HIV infection.

Here, we aimed to identify a metabolomic signature of MetS in HIV-infected individuals to reveal key aspects of the underlying pathophysiology. This may help to improve the understanding of the pathogenesis of this disease and potentially identity new targets for the treatment of this condition and provide better quality of life including healthy aging. To our knowledge, this is the first study to apply several conventional and advanced bioinformatics algorithms to analyze the metabolic alterations associated with MetS in PLWH using untargeted metabolomics.

## RESULTS

### Clinical characteristics

PLWH was stratified according to the presence of MetS syndrome (100 individuals with and 100 individuals without MetS). Clinical characteristics of PLWH are summarized in Table 1. All individuals were currently on cART with similar duration of HIV-infection (*p*-value = 0.329) and cART exposure (*p*-value = 0.455). Larger prevalence of CD4 nadir < 200 cells was found among PLWH with MetS [53 (53%) vs. 38 (38%), *p*-value = 0.039].

**Table 1:** Clinical and demographic characteristics.

	**PLWH with MetS**	**PLWH without MetS**	***P*-values**
***N***	100	100	
**Age**	52 (48–61)	52 (47–62)	0.805^*^
**Gender,** Male, *n* (%)	90 (90)	90 (90)	1^**^
**Mode of transmission,** *n* (%)			
Homosexual/bisexual	73 (73)	71 (71)	0.745^**^
Blood transfusion	1 (1)	0	
PWID	0	1 (1)	
Heterosexual	20 (20)	23 (23)	
Other/unknown	4 (4)	5 (5)	
**CD4 at cART Initiation,** cells/μl, median (IQR)	200 (82–340)	280 (168–354)	0.182^*^
**CD4 at sampling,** cells/μl, median (IQR)	691 (538–865)	700 (547–892)	0.865^*^
**CD8 at sampling,** cells/μl, median (IQR)	830 (620–1215)	755 (580–970)	0.152^*^
**Viral load** (<50 copies/mL), *n* (%)	94 (94)	98 (98)	0.127^**^
**Duration of treatment in years,** mean (sd)	13.4 (7)	12.7 (6·1)	0.455^*^
**CD4 nadir** (<200 cells), *n* (%)	53 (53)	38 (38.0)	0.039^**^
**Time since HIV diagnosis,** years, mean (sd)	17.2 (9.5)	16 (8.1)	0.329^***^
**Current cART, yes,** *n* (%)	100 (100)	100 (100)	1^**^
**BMI,** mean (sd)	26 (3)	23 (4)	<0.001^***^
**Hdl,** mmol/l, median (IQR)	0.9 (0.8–1.1)	1.3 (1.1–1.6)	<0.001^*^
**Tgl,** mmol/l, median (IQR)	2.7 (2.1–3.7)	1.3 (1.1–1.7)	<0.001^*^
**Waist,** cm, median (IQR)	102.0 (95.0–105.2)	89.5 (85.0–97.0)	<0.001^*^
**Systolic BP,** mmHg, median (IQR)	138.5 (131.0–145.2)	124.5 (117.8–142.0)	<0.001^*^
**Diastolic BP,** mmHg, median (IQR)	86.0 (79.0–91.2)	79.0 (73.0–85.2)	<0.001^*^

^*^Mann-Whitney *U* test, ^**^Chi-square test, ^***^T-test. Abbreviations: PLWH: people living with HIV; MetS: metabolic syndrome; PWID: people who inject drugs; IQR: interquartile range; BMI: body mass index; Tgl: triglycerides; BP: blood pressure; cART: combination antiretroviral therapy; sd: Standard deviations.

### Impaired amino acid metabolism in PLWH with MetS

**Figure 1:**
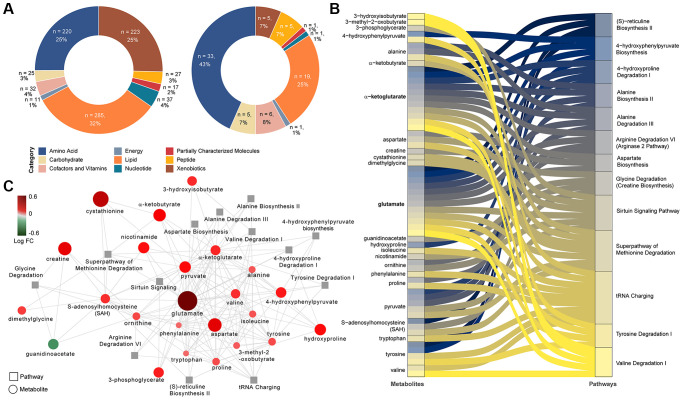
**Differing metabolites and pathways found between PLWH and PLWH with MetS.** (**A**) Doughnut charts of metabolite proportions for each super pathway for all detected metabolites (left) and metabolites with differential abundance between PLWH and PLWH with MetS (LIMMA, FDR < 0.1, *n* = 69). (**B**) Metabolites contribution to the flow of top 13 pathways represented as Sankey Plot. (**C**) Cytoscape network of top 13 pathways and associated enriched metabolites.

The untargeted metabolomics identified 877 characterized biochemicals linked mainly with lipid metabolism (*n* = 285, 32%), xenobiotics including food components (*n* = 223, 25%) and amino acid metabolism (*n* = 220, 25%) (Figure 1A). The relative standard deviation (RSD) for the internal standards for the process variability was 5%. We observed that a total of 76 biochemicals differed significantly between PLWH with and without MetS (Mann-Whitney *U*, FDR <0.05) of which the majority were amino acids (*n* = 33, 43%) (Figure 1A). We next used metabolite set enrichment analysis (MSEA) and network analysis to identify MetS related mechanisms in PLWH and key biochemicals. The MSEA (FDR < 0.1 KEGG and HMDB) identified several affected amino acid metabolic pathways including methionine degradation, valine degradation, tyrosine degradation along the sirtuin signaling pathway that are linked to age-related diseases (Figure 1B). The network analysis identified two key molecules that interact with other metabolites, glutamate, and α-ketoglutarate, that were linked with most of the altered pathways (Figure 1C).

### A combination of methodologies emphasizes the role of glutamate metabolism in MetS among PLWH

**Figure 2:**
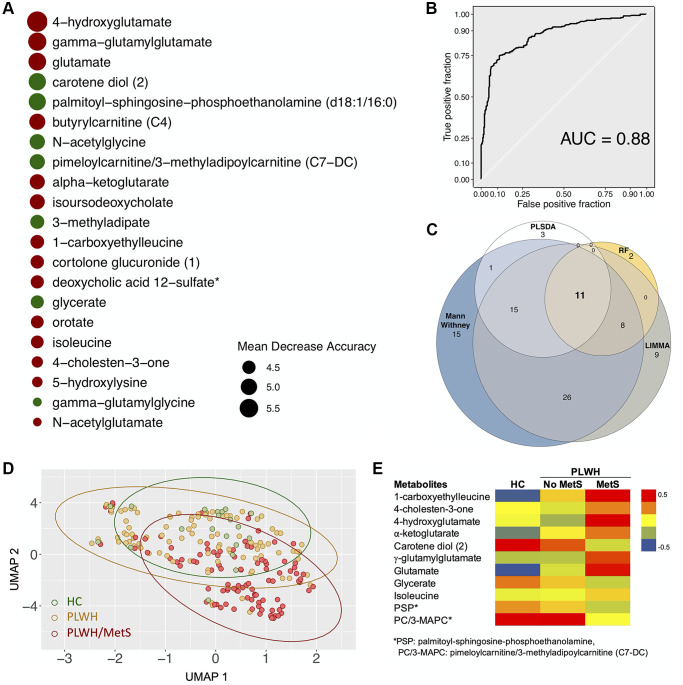
**Biomarkers (*n* = 11) with differential abundance between PLWH and PLWH with MetS identified by four methodologies.** (**A**) Bubble plot representing Random forest variable importance based on mean decrease accuracy (a measure of the model’s performance without each metabolite). Values are scaled by the standard error of the measure. Metabolites represented at the top of the figure are the most important for prediction. (**B**) Receiver Operating Characteristic (ROC) curve of random forest classifier. (**C**) Venn diagram summarizing biomarkers identified by Mann-Whitney *U* test, LIMMA, Random Forest (RF), and PLS-DA. (**D**) UMAP visualization of the 11 biomarkers. Controls (green) and PLWH (yellow) are segregating from PLWH with MetS (red). (**E**) Heatmap showing log2 intensities of the 11 biomarkers in HC, PLWH without MetS and PLWH with MetS.

By combining supervised and unsupervised statistical and machine learning approaches, we sought to identify the most conservative and consensus set of metabolites associated with MetS in PLWH. Then, random forest (RF) was run on data with two classes: PLWH with and without MetS. The top-ranking metabolites (*n* = 21, using the feature selection by Boruta) (Figure 2A) showed several metabolites such as 4-hydroxyglutamate, γ-glutamylglutarate, glutamate, α-ketoglutarate, γ-glutamylglycine and N-acetylglutamate that highlights the central role of glutamate metabolism in MetS. This model achieved 80% predictive accuracy, sensitivity and specificity of 0.8 (Supplementary Figure 1), and an AUROC of 0.88 (Figure 2B). PLS-DA was also used to classify the whole data set (*n* = 877) that was submitted after scaling. A model using the two first components showed a significant coefficient of determination (R2 = 0.58) and coefficient of prediction (Q2 = 0.2). Using the predictive Variable Importance in Projection (VIP) vector, the top 30 crucial metabolites for this model were extracted (Supplementary Figure 2). LIMMA identified 69 metabolites that were significantly different between the groups (FDR < 0.05). A consensus identification among the four methods identified 11 biomarkers that were robustly identified by all approaches (Figure 2C and Supplementary Figure 3). A UMAP projection of samples based on these 11 biomarkers showed a clear separation of the uninfected controls and PLWH with MetS (Figure 2D). Seven of our potential biomarkers were upregulated in PLWH with MetS: 1-carboxyethylleucine, 4-cholesten-3-one, 4-hydroxyglutamate, α-ketoglutamate, γ-glutamylglutamate, glutamate, and isoleucine, while four were downregulated: carotene diol(2), glycerate, PSP and PC/3-MAPC (Figure 2E). γ-glutamylglycine and orotate were found only in RF while N2,N5-diacetylornithine, γ-glutamyl-alpha-lysine and γ-glutamyltyrosine were identified only by PLS-DA. Though our study population was highly matched, majority of the PLWH were male and MetS categorizations varied between male and female. We also perform sensitivity analysis restricted only to the males. Among the metabolites, 13 were identified as consensus among all the analysis (Supplementary Figure 4A). The RF model showed slightly lower performance than the full model (AUROC = 0·85, Supplementary Figure 4B). Interestingly, among the 13 potential biomarkers identified by the same process in males, eight were shared with biomarkers identified in the entire sample population and are mainly linked with glutamate metabolism (Supplementary Figure 4C). The five other biomarkers of MetS in males were γ-glutamylvaline, N-acetylglutamate, N-acetylvaline, γ-glutamylisoleucine and valine (Supplementary Figure 4D) with an increase in PLWH with MetS indicating the altered glutamate metabolism as a common feature in PLWH with MetS. Despite metabolic changes associated with MetS, confirmation that our potential biomarkers were specific for PLWH with MetS and not biomarkers of obesity or treatment was necessary.

We thus tested whether there was an association between the biomarkers and MetS status corrected for BMI. All biomarkers were significant (FDR < 0.05) after BMI and treatment correction (Supplementary Tables 1 and 2). Pharmaceuticals can also affect metabolic profiles. Five xenobiotics were found to be significantly different between PLWH with and without MetS: hydrocinnamate, abacavir, losartan, tartronate, and thioproline. Correction for xenobiotics level was performed for each metabolite using linear regression (Supplementary Table 3). All metabolites passed the correction except glycerate. After correlation analysis, glycerate was shown to have a significant correlation with tartronate (R = 0.8, FDR < 0·00001) (Supplementary Figure 5).

### Impaired central carbon metabolism with higher efflux of key carbohydrates

**Figure 3:**
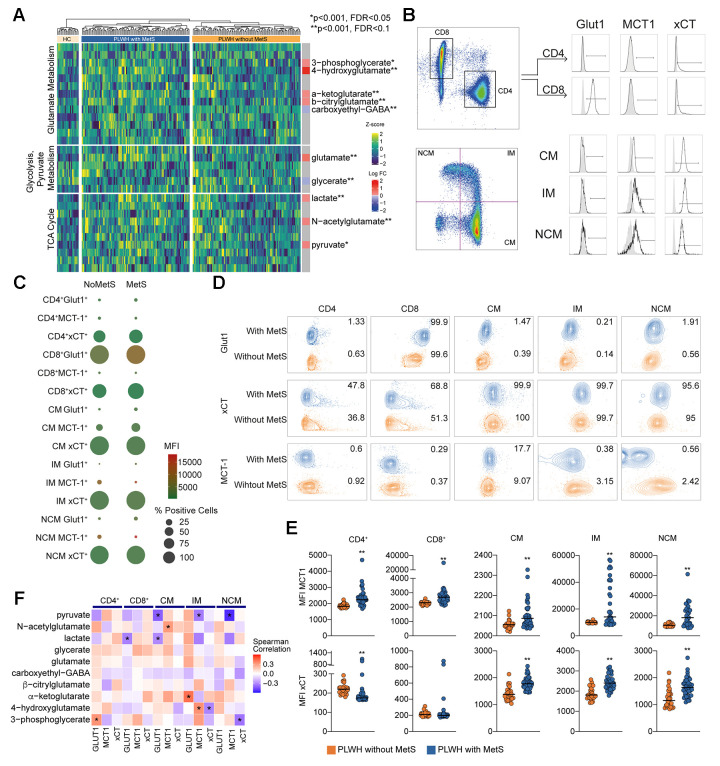
**Central carbon metabolism with higher efflux of key metabolites.** (**A**) Heatmap showing the level of metabolites of glutamate metabolism, glycolysis/gluconeogenesis/pyruvate metabolism, and TCA cycle. The statistically significant differentially abundant metabolites are marked with single asterisk at the level of *p* < 0.001 and FDR < 0.1 and double asterisk *p* < 0.001 and FDR < 0.05 using LIMMA. Single asterisks indicate statistically significant differences *p* < 0.001 and FDR < 0.1 and double asterisk *p* < 0.001 and FDR < 0.05. (**B**) Gating strategy for Glut1, MCT-1, and xCT in T cells (CD4 and CD8) and monocytes (CM, IM, and NCM). (**C**) Bubble plot of Glut1, MCT-1, and xCT in subpopulations. Size of the bubble represents proportion of positive cells (%). Color of the bubbles represent MFI. (**D**) Contour plots showing sample with median percentage of cells for each population. (**E**) MFI for MCT-1 and xCT in lymphocytes (CD4 and CD8) and monocytes (CM, IM, and NCM). (**F**) Co-relation analysis between the transporter expression and the differentially altered metabolites between PLWH with MetS and without MetS as shown in Figure 3A. Asterisk indicates *p* < 0.05.

Higher levels of plasma glutamate and altered glutamate metabolism may indicate dysregulation of central carbon metabolism (CCM). Therefore, we investigated key metabolites from other pathways of central carbon metabolism: glycolysis, pyruvate metabolism, and the TCA cycle (Figure 3A). No changes were observed in plasma glucose levels, but we found a significant increase in pyruvate, lactate, and α-ketoglutarate levels in PLWH with MetS compared to PLWH without MetS (Figure 3A). This indicates an increased efflux of glycolytic and TCA cycle metabolites from cells into the bloodstream in PLWH with MetS. Furthermore, to characterize the uptake and secretion profile of key metabolic transporters within CCM, we measured the expression of Glut1 (glucose transporter), MCT-1 (pyruvate and lactate transporter), and xCT (transporter exchanging glutamate for cystine) in a subset of samples using flow cytometry (Figure 3B). We observed a significant decrease in classical monocytes in PLWH with MetS compared to PLWH without MetS while no difference was observed in other PBMC subpopulations (Supplementary Figure 6A). Most differences seen between the groups were on xCT and MCT-1 expression (Figure 3C). Percentage of cells expressing the different metabolite transporters were altered in PLWH with MetS compared to PLWH without MetS (Figure 3D). For Glut1, the median fluorescence intensity (MFI) was significantly higher in CD8^+^ T-cells and non-classical monocytes in PLWH with MetS compared to without MetS (Supplementary Figure 6B). PLWH with MetS had significantly increased expression of MCT-1 on all sub-populations of PBMCs compared to without MetS (Figure 3E). For xCT, expression levels were significantly increased in all monocytic cells (classical, intermediate, and non-classical) and decreased in CD4^+^ T-cells in PLWH with MetS compared to without MetS. These results indicate that PLWH with MetS had higher metabolic uptake and secretion in monocytic cell subsets compared to PLWH without MetS, specifically for glutamate transport across the cell membrane. The correlation between the transporter expression (represented as MFI) and significantly different metabolites in PLWH with MetS indicates negative co-relation of monocytes subpopulations of Glut1 with pyruvate level and CD8^+^ T-cells and classical monocytes with lactate level (Figure 3F). These further support altered metabolic state of the cells in the PLWH with MetS.

### Community analyses confirm identified biomarkers and display possible mechanisms of metabolic syndrome in PLWH

**Figure 4:**
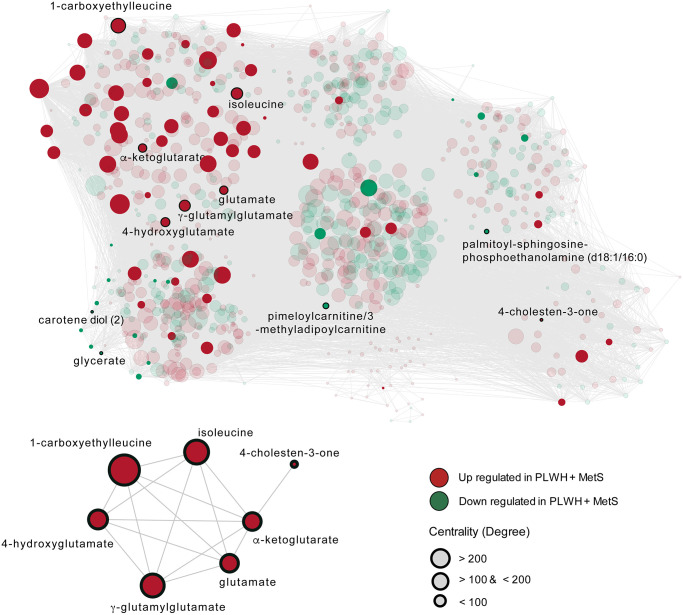
**The metabolome-wide weighted co-expression network.** A weighted metabolite co-expression network was generated (positive Spearman rank correlations, FDR < 0·05). Significant metabolites based on LIMMA have represented opaques and non-significant transparent. Biomarkers found in the first community are represented (bottom-left).

To establish the metabolic shifts (i.e., the metabolic alterations due to disease conditions) associated with MetS in PLWH, a weighted co-expression network analysis was performed using all detected metabolites and all patients. In this network, the nodes were individual metabolites, and edges were modeled by the positive coefficients of correlation between two metabolites (Spearman, FDR < 0·05). The resulting network consisted of 867 nodes and 45379 edges. Leiden partitioning highlights seven communities of positively intercorrelated metabolites (Figure 4 top). Analysis at the pathway level revealed that the most central community, represented as the center of the network, was enriched for the KEGG terms 2-oxocarboxylic acid metabolism, organic acids, and lipids (Metabolon™ category). This community displayed a very heterogeneous pattern, where only six differentially expressed metabolites (out of 73) were present and half had higher abundance in PLWH with MetS. Interestingly, we observed that a single community contained six potential biomarkers previously identified (Figure 4 bottom). This community also displayed 38 differentially expressed metabolites, of which only one was lower abundant in PLWH with MetS and contained six of the 11 potential biomarkers identified above. These biomarkers showed many interconnections between each other, though other biomarkers including 4-cholesten-3-one were segregated in other communities. This community was enriched in common amino acids (KEGG and Metabolon) and peptides (Metabolon).

## DISCUSSION

In this study, using a consensus approach of traditional biostatistics and advanced machine learning algorithms, we first identified altered amino acid metabolism as a central characteristic of PLWH with MetS with glutamate metabolism as a key metabolic pathway in this phenotype. Second, a weighted co-expression network analysis indicated the interaction between lipid metabolism and amino acid metabolism that could modulate glutamate metabolism as a coordinated metabolic alteration in PLWH with MetS. Finally, immune phenotyping of the transporter expression identified that both lymphocytic and monocytic cells exhibited increased levels of glutamate to cysteine transport, indicating elevated glutaminolysis in PLWH with MetS compared to PLWH without MetS.

The role of amino acid metabolism in the development of metabolic dysfunction has been extensively studied in the general population, but it is still unclear in the context of HIV infection. The most consistent findings describe an essential role for the three branched-chain amino acids (BCAA) (leucine, isoleucine, and valine) in the development of cardiometabolic diseases, in particular, obesity and diabetes [10]. In our study, we observed higher levels of isoleucine associated with MetS among PLWH. This finding aligns with previous studies describing increased isoleucine levels in nascent metabolic syndrome in the general population [11]. All three BCAA are catabolized into glutamate and a branched-chain-ketoacid (BCKA) via the transamination of α-ketoglutamate by branched-chain aminotransferase (BCAT). BCKA is further metabolized by the activity of the enzyme branched-chain-ketoacid dehydrogenase (BCKD). Alterations of BCAT and BCKD activity have been described in individuals with MetS syndrome [12]. In particular, lower expression of BCKD was described in visceral, but not subcutaneous, adipose tissue of metabolically impaired subjects, leading to reduced activity of this metabolic pathway and consequent accumulation of BCAA and glutamate. Accordingly, glutamate concentrations have been efficiently used to differentiate individuals with an excess of adipose tissue at abdominal level and metabolic risk [13]. Taken together these findings suggest a potential role for alterations of BCAA catabolism in abdominal adipocytes in the pathogenesis of MetS in the background population. Previous results from our group proposed excess abdominal adipose tissue as a critical factor in the excess risk of MetS in the context of HIV compared to uninfected individuals [4]. The identification of metabolome alterations in PLWH with MetS may help to explain this not yet wholly understood association. In the present study, increased levels of several metabolites involved in glutamate metabolism characterized PLWH with MetS. This finding may point towards a significant association between glutamate and MetS among PLWH, thus supporting previous studies in uninfected individuals [11]. Given the previously described association of HIV infection with alterations of both abdominal obesity [4] and glutamate metabolism [14, 15], one may speculate that the role of glutamate metabolism in the pathogenesis of MetS might be even more central in the context of HIV.

In the present study, PLWH both with and without MetS had higher concentrations of several metabolites involved in glutamate metabolism compared to uninfected controls. Although the sample size of uninfected controls is small, these findings support a previous study that showed higher glutamate in PLWH than uninfected controls [14]. The association between HIV infection and glutamate metabolism has also been studied in the context of neurologic comorbidities [16]. Accordingly, increased release of glutamate in the extracellular space by HIV-infected macrophages mediated by the viral protein Vpr has been suggested to play a central role in the pathogenesis of HIV-mediated neurotoxicity [17]. It is to be noted that both lactate and pyruvate were also increased in the PLWH with cART and PLWH without MetS. It is known that lactate and pyruvate reduced glutamate-induced neurotoxicities in animal experiments [18]. One may speculate that increased lactate and pyruvate could be due to the increased glutamate thereby linked with the severity of the MetS due to impaired aerobic metabolism and elevated metabolic diseases in PLWH. This is further supported by the altered expression of xCT and MCT-1 in the blood cell populations. However, this may have a protective effect on neurological impairment [19]. However, whether perturbed glutamate homeostasis is also involved in other non-AIDS-associated comorbidities like MetS is still unknown. Abdominal adipose tissue accumulation, a well-known determinant of MetS in PLWH, has been previously suggested to be associated with increase macrophage infiltration and activation [20]. It may be speculated that alterations in macrophage glutamate metabolism in the abdominal tissue are also involved in MetS in PLWH. Thus, novel treatments targeted at reducing plasma glutamate concentration could potentially be implemented along with cART to prevent both neurological and metabolic complications in PLWH with MetS and increased build-up of lactate and pyruvate, also known for toxic properties [21].

Moreover, HIV-1 induces oxidative stress by increasing the reactive oxygen species (ROS) production mediated by the HIV-1 proteins [22] and *in vitro* studies showed that the inhibition of glutaminolysis affects ROS homeostasis in cancer cells [23]. Further, glutaminolysis activates mammalian target of rapamycin (mTOR) is a key component of cellular metabolism which plays an essential role in the age related processes. [24]. Studies also have reported that mTOR complex modulates HIV-1 latency [25], and that inhibition of the mTOR complex 1 (mTORC1) inhibits HIV-1 replication and suppresses latency reversal [26]. Therefore, a glutamatergic drug that directly modulates the excitatory glutamate in the body or brain can potentially be used after appropriate clinical studies. It can act as novel strategies both for healthy-aging in PLWH and functional cure by elimination of the latent HIV-reservoir during the successful cART.

The present study has several limitations. First, due to the cross-sectional design, no conclusions on causality can be drawn. Second, the relatively low number of uninfected individuals were included and the lack of clinical data in this population prevented us from investigating the impact of HIV infection on the metabolites and pathways that are observed to be associated with MetS. Also, the uninfected controls were used to identify the metabolites’ normal range as untargeted metabolomics analysis is dependent upon the run, thus not used for any statistical analysis. Finally, despite adjusting for multiple testing, the high number of associations tested may have led to type I errors. However, the major strength of this study is the use of relatively larger, well-characterized, clinically matched cohorts of PLWH. To the best of our knowledge, this is the first study investigating metabolome alterations associated with MetS in the context of HIV-infection.

In conclusion, our data on changes in the plasma metabolomics profile and altered expression of the metabolite transporters in blood cells suggesting alterations in glutamate metabolism to be associated with MetS in PLWH. Glutamate plays a central role in multiple metabolic pathways for the interchange of amino nitrogen by both amino acid synthesis as well as degradation, and glutamate toxicity is associated with age-related neurodegenerative disorders. We further hypothesize that metabolic stress could be the reason for accelerated aging in PLWH with MetS, which needs clinical interventions to improve the metabolic profile to provide a better quality of life. Further studies are warranted to address a possible direct role of glutamate in the pathogenesis of MetS and its potential role as a biomarker for accelerated cognitive and metabolic aging in the context of HIV-infection.

## MATERIALS AND METHODS

### Study population

PLWH were recruited from the Copenhagen Comorbidity in HIV-infection (COCOMO) study, an ongoing longitudinal, observational study to assess the burden of non-AIDS comorbidities in HIV infection. Of the 1099 participants in the COCOMO study, 100 PLWH with MetS were randomly selected and matched to 100 PLWH without MetS. Individuals were matched according to age, sex, duration of cART, smoking status, and current CD4^+^ T-cells count. All plasma samples were collected at the study baseline concomitant with clinical assessment. Given that untargeted metabolomics is a relative measurement of the metabolites we included HIV-negative controls (*n* = 20). They were also included in this study to define the level of metabolites considered as normal range in the assay run. Procedures for recruitment and data collection for COCOMO have been described elsewhere [27]. Ethical approval was obtained by the Regional Ethics Committee of Copenhagen (COCOMO: H-15017350). Written informed consent was obtained from all participants.

### Clinical and biochemical assessments

Structured questionnaires were used in COCOMO to collect information about demographics, physical activity, smoking, lipid-lowering, and antihypertensive therapy [27]. Data regarding HIV-infection were obtained from a review of medical charts [27]. All physical examinations were performed by trained clinic staff, as previously described [27]. Height, weight, hip, and waist measurements and body mass index (BMI) calculations were performed according to WHO guidelines. Blood pressure (BP) was measured on the left arm after 5 minutes rest with the subject in sitting position, using an automatic Digital Blood Pressure Monitor. Non-fasting venous blood was collected and analyzed for LDL-C, total cholesterol, HbA1c, and glucose. Blood samples were analyzed at Herlev Hospital, Copenhagen. Metabolic syndrome (MetS) was defined according to the International Diabetes Federation (IDF) consensus worldwide definition of the MetS [28] as ≥3 of the following: (1) waist circumference ≥94 cm in men and ≥80 cm in women, (2) systolic blood pressure ≥130 mm Hg and/or diastolic blood pressure ≥85 mm Hg and/or antihypertensive treatment, (3) non-fasting plasma triglyceride level ≥1.693 mmol/L, (4) HDL level ≤1.036 mmol/L in men or ≤0.295 mmol/L in women, and (5) self-reported diabetes and/or antidiabetic treatment and/or plasma glucose level ≥11.1 mmol/L.

### Sample preparation and untargeted metabolomics

Untargeted metabolite profiling was carried out by Metabolon Inc. (Durham, NC, USA) using ultra-high-performance liquid chromatography/mass spectrometry/mass spectrometry (UHPLC/MS/MS) as described earlier [15, 29]. Data was normalized to sample volume, log-normalized, and minimum-imputed as given by the proprietary pipeline of the provider. The metabolomics method is ISO 9001:2015 certified and the lab is accredited by the College of American Pathologists (CAP), USA.

### Statistics and bioinformatics analysis

For clinical data, Welch’s *T*-test and Mann-Whitney *U* test were used to compare normally distributed and non-normally distributed continuous variables, respectively. Chi-Square Test was used to compare discrete variables if expected values of the contingency table were five or more. Otherwise, Fisher’s Exact Test was used. For metabolomics data, the median-centered data was used for all the analyses. To define metabolic alterations of the MetS in PLWH, we used a consensus of four different algorithms as the predictive signatures of MetS in PLWH. We used two conventional approaches LIMMA and Mann-Whitney *U* test., and one machine learning algorithm [random forest (RF)], and one linear classification model (PLS-DA) to identify the most important metabolites associated with MetS in PLWH using R packages ropls and RF, respectively [30]. Both the models separate the groups, based on the importance of metabolites. The top 30 most important variables from the PLS-DA were extracted using VipPlot [31]. For RF, feature selection was performed using the R package Boruta. A RF model using 10-cross fold validation was built using selected metabolites. Performance comparison of models was done using confusion matrix, ROC curves and area under the curve (AUC). As a sensitivity analysis, a similar analysis was performed using only male patients. To identify the consensus biomarkers, overlap of methodologies was represented as a Venn diagram using the R package eulerr ing ggplot2. Adjustment for multiple testing was performed by considering false discovery rate (FDR) <0.05.

### Identification of mechanistic pathways

To highlight the most enriched pathways in PLHW with MetS group compared to PLHW without MetS, metabolites with differential abundance (LIMMA, FDR<0.05) were submitted to Ingenuity Pathway Analysis software (IPA) (Qiagen, US). The top 13 enriched pathways and associated metabolites were extracted, represented as a Sankey plot using R package ggalluvial and network using Cytoscape version 3.6.1 [32]. Metabolite-metabolite interactions with high confidence (confidence >0.7) were retrieved from databases and experiments uploaded to STITCH(v5.0) (http://stitch.embl.de/). Association analysis was performed using R and python 3. First, metabolites with low variance were removed. Then, pairwise Spearman correlations between all metabolites were performed. Only correlations with FDR<0.05 were conserved. The distribution of correlation coefficients was plotted to evaluate the size and connection strength of the networks. Accordingly, a weighted co-expression network for significant positive associations for all samples was constructed using the python module igraph (https://igraph.org/python/). Random networks with the same dimensions were also constructed and network properties analyzed. Leiden algorithm was used for partitioning the network using the python module leidenalg [33]. The average degree and clustering coefficient were computed for all communities and functional enrichment was performed for more than 30 metabolites. Metabolite set enrichment analysis (MSEA) was performed on these communities using gseapy first with an in-house script containing Metabolon terms then KEGG terms. Communities partition and network were exported from python and imported to Cytoscape. Metabolites with significant differential abundance between PLWH without MetS and PLWH with MetS based on LIMMA, as well as biomarkers identified by the four methodologies were highlighted in the global network.

### Flow cytometry

In a subset of samples PLWH with MetS (*n* = 31) and without MetS (*n* = 27) we performed immunophenotyping of peripheral blood mononuclear cells (PBMCs) for transporter expression of glucose transporter (Glut1), monocarboxylic acid transporters (MCT-1), and glutamate/cystine transporter xCT on T-cells (CD4^+^ and CD8^+^) and monocytes (classical, intermediate and non-classical) as described by us recently [29]. All cells were stained with Live/Dead fixable near IR dye (Invitrogen), and cell surface markers were detected by incubating cells with relevant antibodies for 20 min on ice in flow cytometry buffer (antibodies listed in Supplementary Table 4). Cells were acquired a BD FACS Symphony flow cytometer (BD Bioscience), and data were analyzed and compensated with FlowJo 10.7 (TreeStar Inc), and Prism 8 (GraphPad Software Inc).

## Supplementary Materials

Supplementary Figures

Supplementary Tables

## References

[1] van Sighem AI, Gras LA, Reiss P, Brinkman K, de Wolf F, and ATHENA national observational cohort study. Life expectancy of recently diagnosed asymptomatic HIV-infected patients approaches that of uninfected individuals. AIDS. 2010; 24:1527–35. 10.1097/QAD.0b013e32833a3946 20467289

[2] Petoumenos K, Reiss P, Ryom L, Rickenbach M, Sabin CA, El-Sadr W, d'Arminio Monforte A, Phillips AN, De Wit S, Kirk O, Dabis F, Pradier C, Lundgren JD, Law MG, and D:A:D study group. Increased risk of cardiovascular disease (CVD) with age in HIV-positive men: a comparison of the D:A:D CVD risk equation and general population CVD risk equations. HIV Med. 2014; 15:595–603. 10.1111/hiv.12162 24840675

[3] Sackoff JE, Hanna DB, Pfeiffer MR, Torian LV. Causes of death among persons with AIDS in the era of highly active antiretroviral therapy: New York City. Ann Intern Med. 2006; 145:397–406. 10.7326/0003-4819-145-6-200609190-00003 16983127

[4] Gelpi M, Afzal S, Lundgren J, Ronit A, Roen A, Mocroft A, Gerstoft J, Lebech AM, Lindegaard B, Kofoed KF, Nordestgaard BG, Nielsen SD. Higher Risk of Abdominal Obesity, Elevated Low-Density Lipoprotein Cholesterol, and Hypertriglyceridemia, but not of Hypertension, in People Living With Human Immunodeficiency Virus (HIV): Results From the Copenhagen Comorbidity in HIV Infection Study. Clin Infect Dis. 2018; 67:579–86. 10.1093/cid/ciy146 29471519

[5] Gelpi M, Vestad B, Hansen SH, Holm K, Drivsholm N, Goetz A, Kirkby NS, Lindegaard B, Lebech AM, Hoel H, Michelsen AE, Ueland T, Gerstoft J, et al. Impact of Human Immunodeficiency Virus-Related Gut Microbiota Alterations on Metabolic Comorbid Conditions. Clin Infect Dis. 2020; 71:e359–67. 10.1093/cid/ciz1235 31894240

[6] Zhang XW, Li QH, Xu ZD, Dou JJ. Mass spectrometry-based metabolomics in health and medical science: a systematic review. RSC Advances. 2020; 10:3092–04. 10.1039/C9RA08985C PMC9048967 35497733

[7] Bogdanov M, Matson WR, Wang L, Matson T, Saunders-Pullman R, Bressman SS, Flint Beal M. Metabolomic profiling to develop blood biomarkers for Parkinson's disease. Brain. 2008; 131:389–96. 10.1093/brain/awm304 18222993

[8] Hirayama A, Kami K, Sugimoto M, Sugawara M, Toki N, Onozuka H, Kinoshita T, Saito N, Ochiai A, Tomita M, Esumi H, Soga T. Quantitative metabolome profiling of colon and stomach cancer microenvironment by capillary electrophoresis time-of-flight mass spectrometry. Cancer Res. 2009; 69:4918–25. 10.1158/0008-5472.CAN-08-4806 19458066

[9] Sreekumar A, Poisson LM, Rajendiran TM, Khan AP, Cao Q, Yu J, Laxman B, Mehra R, Lonigro RJ, Li Y, Nyati MK, Ahsan A, Kalyana-Sundaram S, et al. Metabolomic profiles delineate potential role for sarcosine in prostate cancer progression. Nature. 2009; 457:910–14. 10.1038/nature07762 19212411 PMC2724746

[10] Lent-Schochet D, McLaughlin M, Ramakrishnan N, Jialal I. Exploratory metabolomics of metabolic syndrome: A status report. World J Diabetes. 2019; 10:23–36. 10.4239/wjd.v10.i1.23 30697368 PMC6347655

[11] Shim K, Gulhar R, Jialal I. Exploratory metabolomics of nascent metabolic syndrome. J Diabetes Complications. 2019; 33:212–16. 10.1016/j.jdiacomp.2018.12.002 30611573

[12] Lackey DE, Lynch CJ, Olson KC, Mostaedi R, Ali M, Smith WH, Karpe F, Humphreys S, Bedinger DH, Dunn TN, Thomas AP, Oort PJ, Kieffer DA, et al. Regulation of adipose branched-chain amino acid catabolism enzyme expression and cross-adipose amino acid flux in human obesity. Am J Physiol Endocrinol Metab. 2013; 304:E1175–87. 10.1152/ajpendo.00630.2012 23512805 PMC3680678

[13] Maltais-Payette I, Allam-Ndoul B, Pérusse L, Vohl MC, Tchernof A. Circulating glutamate level as a potential biomarker for abdominal obesity and metabolic risk. Nutr Metab Cardiovasc Dis. 2019; 29:1353–60. 10.1016/j.numecd.2019.08.015 31668457

[14] Scarpellini B, Zanoni M, Sucupira MC, Truong HM, Janini LM, Segurado ID, Diaz RS. Plasma Metabolomics Biosignature According to HIV Stage of Infection, Pace of Disease Progression, Viremia Level and Immunological Response to Treatment. PLoS One. 2016; 11:e0161920. 10.1371/journal.pone.0161920 27941971 PMC5152829

[15] Babu H, Sperk M, Ambikan AT, Rachel G, Viswanathan VK, Tripathy SP, Nowak P, Hanna LE, Neogi U. Plasma Metabolic Signature and Abnormalities in HIV-Infected Individuals on Long-Term Successful Antiretroviral Therapy. Metabolites. 2019; 9:210. 10.3390/metabo9100210 31574898 PMC6835959

[16] Vázquez-Santiago FJ, Noel RJ Jr, Porter JT, Rivera-Amill V. Glutamate metabolism and HIV-associated neurocognitive disorders. J Neurovirol. 2014; 20:315–31. 10.1007/s13365-014-0258-2 24867611 PMC4098898

[17] Datta PK, Deshmane S, Khalili K, Merali S, Gordon JC, Fecchio C, Barrero CA. Glutamate metabolism in HIV-1 infected macrophages: Role of HIV-1 Vpr. Cell Cycle. 2016; 15:2288–98. 10.1080/15384101.2016.1190054 27245560 PMC5004675

[18] Ros J, Pecinska N, Alessandri B, Landolt H, Fillenz M. Lactate reduces glutamate-induced neurotoxicity in rat cortex. J Neurosci Res. 2001; 66:790–94. 10.1002/jnr.10043 11746403

[19] Jones TE, Pories WJ, Houmard JA, Tanner CJ, Zheng D, Zou K, Coen PM, Goodpaster BH, Kraus WE, Dohm GL. Plasma lactate as a marker of metabolic health: Implications of elevated lactate for impairment of aerobic metabolism in the metabolic syndrome. Surgery. 2019; 166:861–66. 10.1016/j.surg.2019.04.017 31253418 PMC7142375

[20] Gelpi M, Ueland PM, Trøseid M, Mocroft A, Lebech AM, Ullum H, Midttun Ø, Lundgren J, Nielsen SD. Abdominal Adipose Tissue Is Associated With Alterations in Tryptophan-Kynurenine Metabolism and Markers of Systemic Inflammation in People With Human Immunodeficiency Virus. J Infect Dis. 2020; 221:419–27. 10.1093/infdis/jiz465 31538186

[21] Zhou C, Sun R, Zhuang S, Sun C, Jiang Y, Cui Y, Li S, Xiao Y, Du Y, Gu H, Liu Q. Metformin prevents cerebellar granule neurons against glutamate-induced neurotoxicity. Brain Res Bull. 2016; 121:241–45. 10.1016/j.brainresbull.2016.02.009 26876755

[22] Ivanov AV, Valuev-Elliston VT, Ivanova ON, Kochetkov SN, Starodubova ES, Bartosch B, Isaguliants MG. Oxidative Stress during HIV Infection: Mechanisms and Consequences. Oxid Med Cell Longev. 2016; 2016:8910396. 10.1155/2016/8910396 27829986 PMC5088339

[23] Goto M, Miwa H, Shikami M, Tsunekawa-Imai N, Suganuma K, Mizuno S, Takahashi M, Mizutani M, Hanamura I, Nitta M. Importance of glutamine metabolism in leukemia cells by energy production through TCA cycle and by redox homeostasis. Cancer Invest. 2014; 32:241–47. 10.3109/07357907.2014.907419 24762082

[24] Durán RV, Oppliger W, Robitaille AM, Heiserich L, Skendaj R, Gottlieb E, Hall MN. Glutaminolysis activates Rag-mTORC1 signaling. Mol Cell. 2012; 47:349–58. 10.1016/j.molcel.2012.05.043 22749528

[25] Besnard E, Hakre S, Kampmann M, Lim HW, Hosmane NN, Martin A, Bassik MC, Verschueren E, Battivelli E, Chan J, Svensson JP, Gramatica A, Conrad RJ, et al. The mTOR Complex Controls HIV Latency. Cell Host Microbe. 2016; 20:785–97. 10.1016/j.chom.2016.11.001 27978436 PMC5354304

[26] Kumar B, Arora S, Ahmed S, Banerjea AC. Hyperactivation of mammalian target of rapamycin complex 1 by HIV-1 is necessary for virion production and latent viral reactivation. FASEB J. 2017; 31:180–91. 10.1096/fj.201600813R 27702769

[27] Ronit A, Haissman J, Kirkegaard-Klitbo DM, Kristensen TS, Lebech AM, Benfield T, Gerstoft J, Ullum H, Køber L, Kjær A, Kofoed K, Vestbo J, Nordestgaard B, et al. Copenhagen comorbidity in HIV infection (COCOMO) study: a study protocol for a longitudinal, non-interventional assessment of non-AIDS comorbidity in HIV infection in Denmark. BMC Infect Dis. 2016; 16:713. 10.1186/s12879-016-2026-9 27887644 PMC5124288

[28] Alberti KG, Zimmet P, Shaw J. Metabolic syndrome--a new world-wide definition. A Consensus Statement from the International Diabetes Federation. Diabet Med. 2006; 23:469–80. 10.1111/j.1464-5491.2006.01858.x 16681555

[29] Sperk M, Mikaeloff F, Svensson-Akusjärvi S, Krishnan S, Ponnan SM, Ambikan AT, Nowak P, Sönnerborg A, Neogi U. Distinct lipid profile, low-level inflammation, and increased antioxidant defense signature in HIV-1 elite control status. iScience. 2021; 24:102111. 10.1016/j.isci.2021.102111 33659876 PMC7892918

[30] Thévenot EA, Roux A, Xu Y, Ezan E, Junot C. Analysis of the Human Adult Urinary Metabolome Variations with Age, Body Mass Index, and Gender by Implementing a Comprehensive Workflow for Univariate and OPLS Statistical Analyses. J Proteome Res. 2015; 14:3322–35. 10.1021/acs.jproteome.5b00354 26088811

[31] Worley B, Powers R. Multivariate Analysis in Metabolomics. Curr Metabolomics. 2013; 1:92–107. 10.2174/2213235X11301010092 26078916 PMC4465187

[32] Shannon P, Markiel A, Ozier O, Baliga NS, Wang JT, Ramage D, Amin N, Schwikowski B, Ideker T. Cytoscape: a software environment for integrated models of biomolecular interaction networks. Genome Res. 2003; 13:2498–504. 10.1101/gr.1239303 14597658 PMC403769

[33] Traag VA, Waltman L, van Eck NJ. From Louvain to Leiden: guaranteeing well-connected communities. Sci Rep. 2019; 9:5233. 10.1038/s41598-019-41695-z 30914743 PMC6435756

