# Supplementary material for: The central role of the glutamate metabolism in long-term antiretroviral treated HIV-infected individuals with metabolic syndrome
Source: Aging (Albany NY). 2021 Oct 11;13(19):22732–51. doi: 10.18632/aging.203622 (PMC8544298; doi:10.18632/aging.203622)
Supplement: Supplementary Figures [file aging-13-203622-s001.pdf]

SUPPLEMENTARY FIGURES

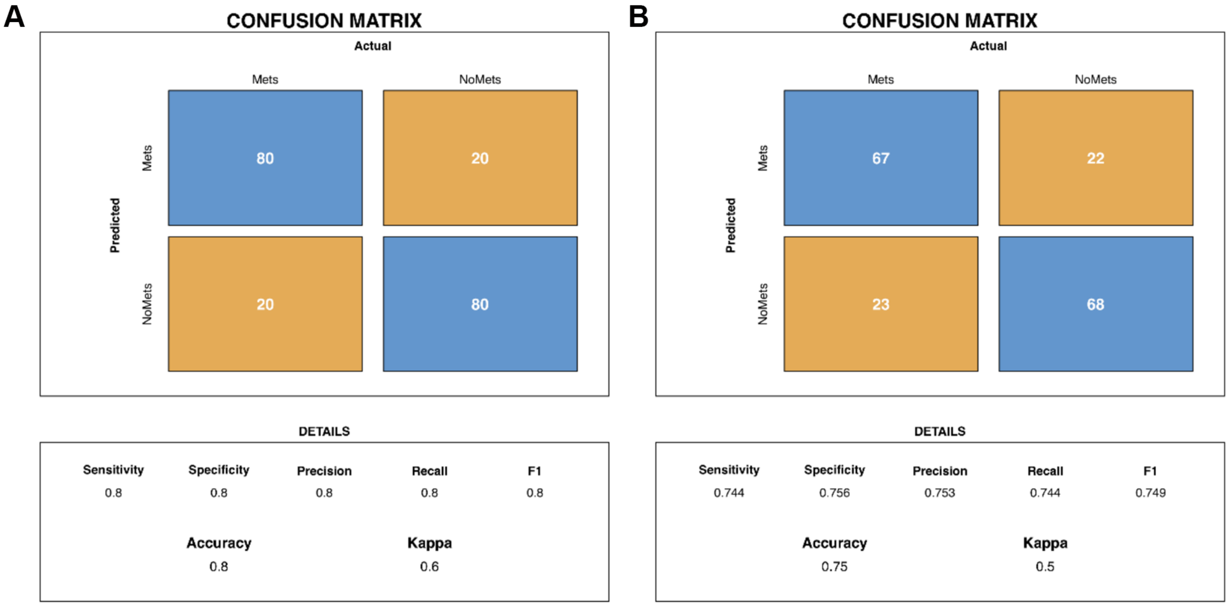

Supplementary Figure 1. Confusion matrices for random forest models (**A**) complete data set (**B**) males only.

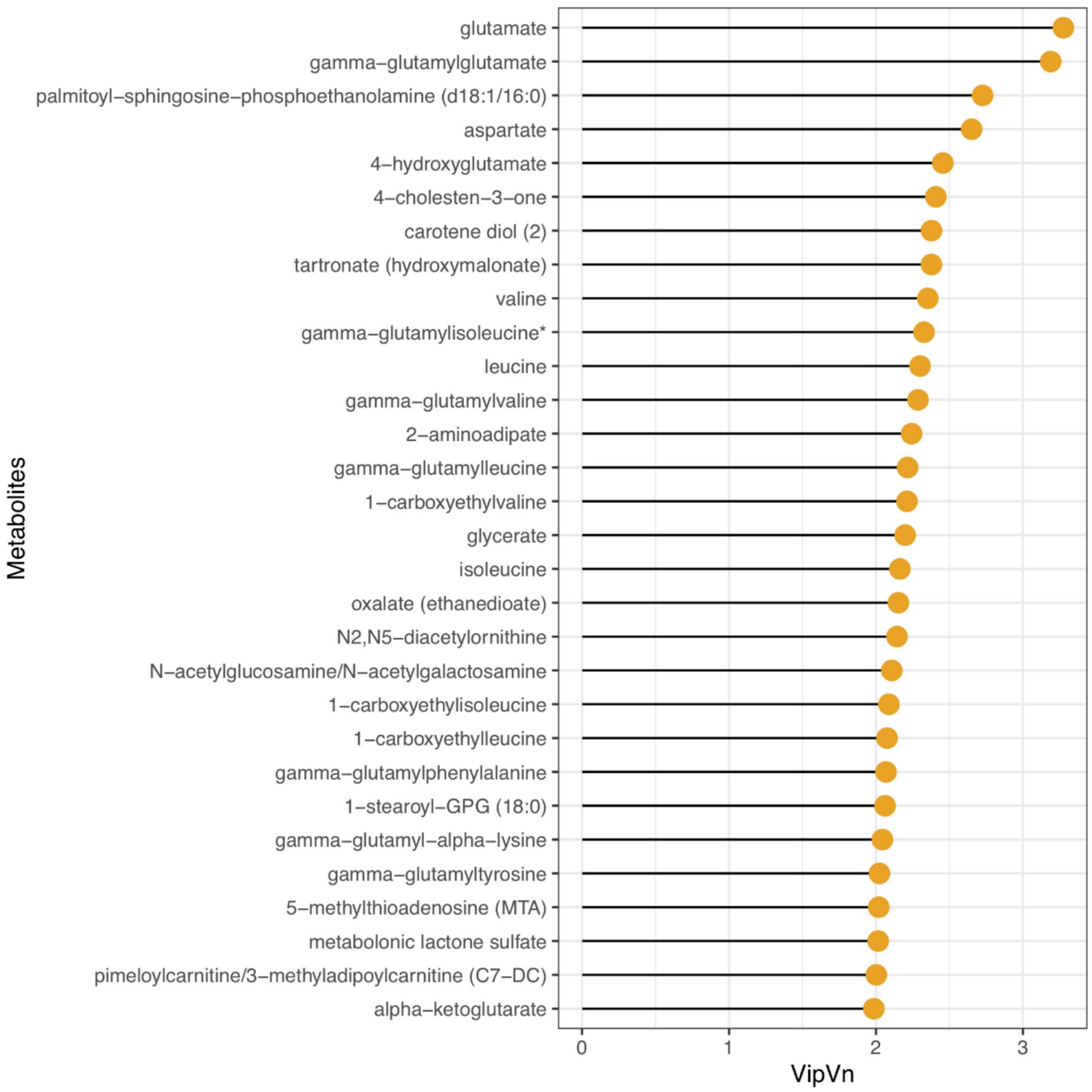

Supplementary Figure 2. Variable importance plot representing top feature importance extracted from PLS-DA model in complete data set.

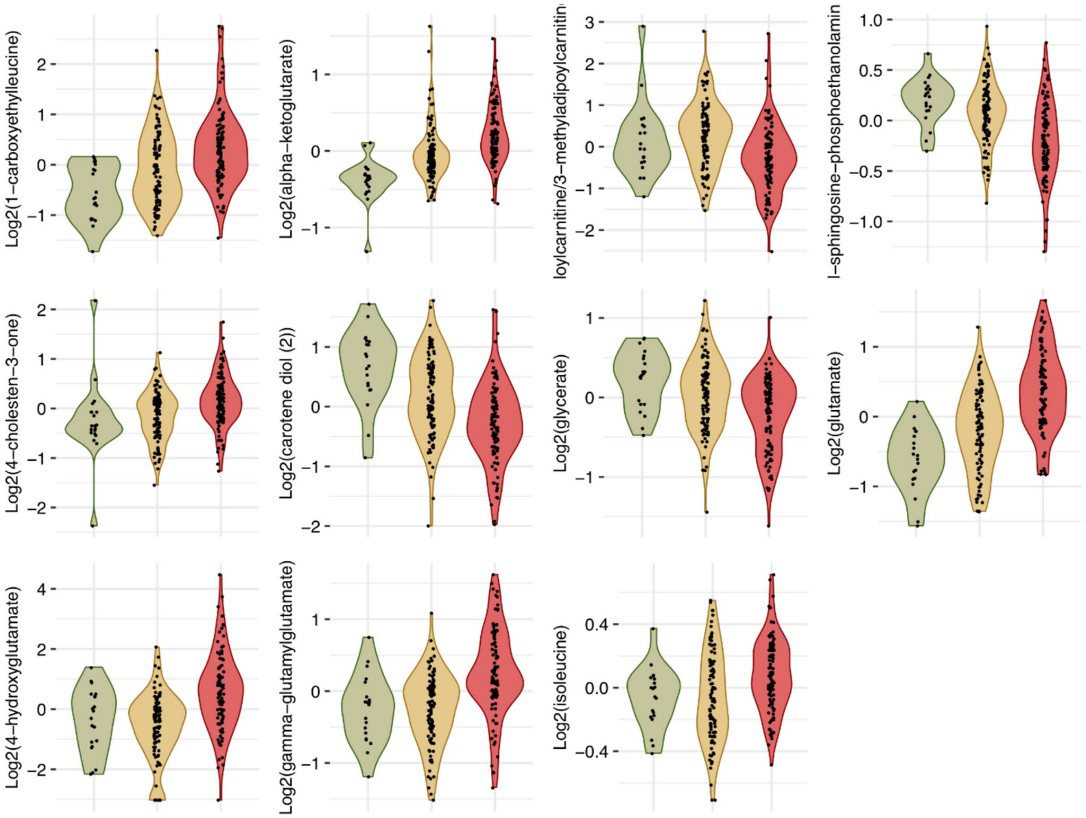

Supplementary Figure 3. Violin plots showing log2 intensities of 11 identified biomarkers in Controls (green), PLWH (yellow) and PLWH+MetS (red) patients.

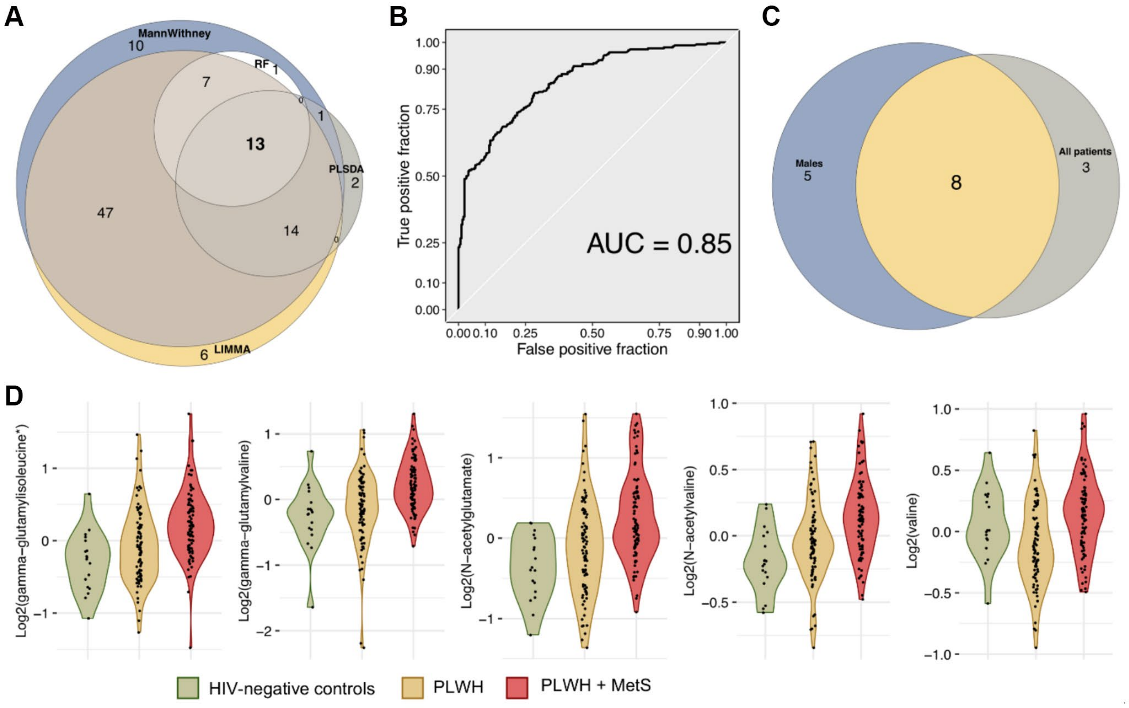

Supplementary Figure 4. Biomarkers with differential abundance between males PLWH and PLWH with MetS. (**A**) Venn diagram summarizing biomarkers identified by Mann-Whitney *U* test, LIMMA, Random Forest (RF) and PLS-DA in male patients. (**B**) ROC curve of random forest classifier for predicting metabolic syndrome status in PLWH in male patients. (**C**) Venn diagram representing the overlap between biomarkers identified in all patients and in males. (**D**) Violin plots showing log2 intensities of 5 identified biomarkers in Controls (green), PLWH (yellow) and PLWH with MetS (red) specific to male patients.

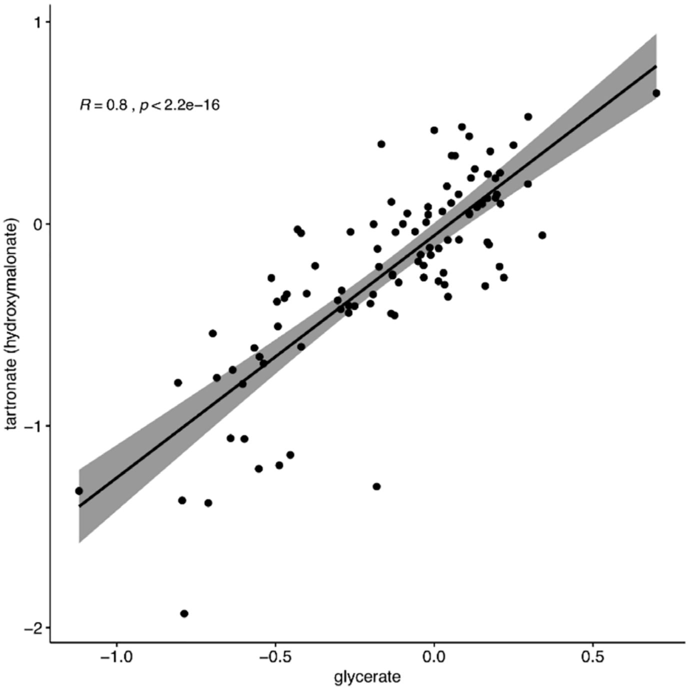

Supplementary Figure 5. Scatter plot representing relationship between scaled intensities of glycerate and tartronate. The regression line, Pearson correlation coefficient and *p*-value are indicated.

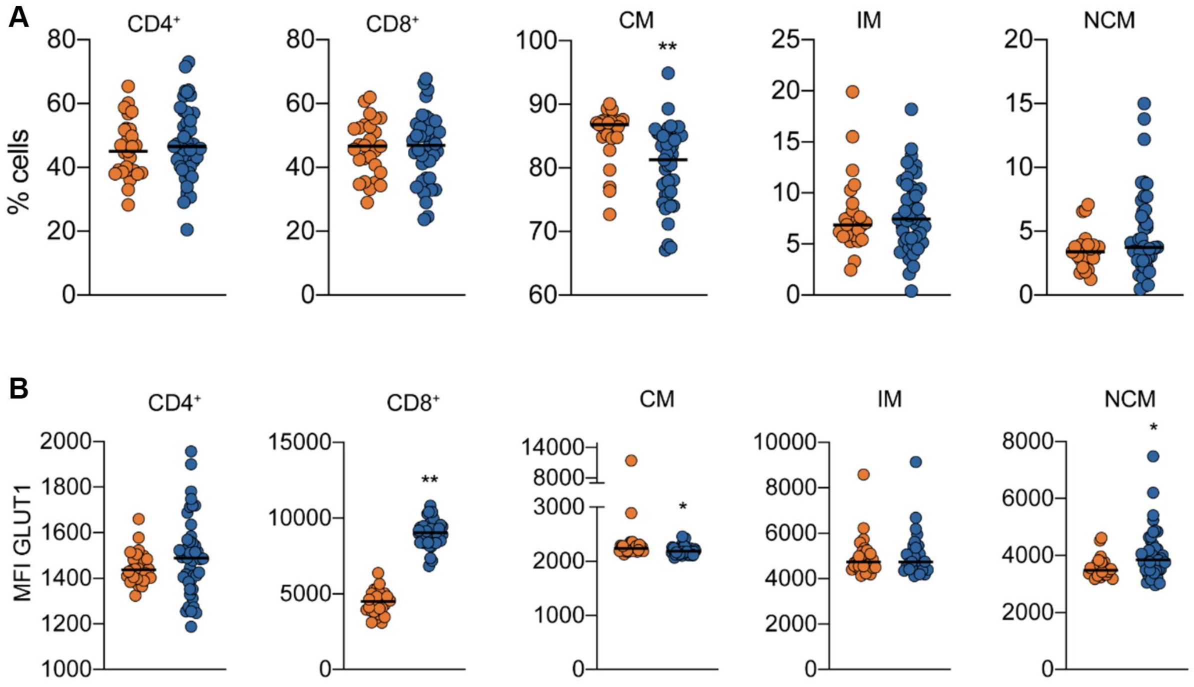

Supplementary Figure 6. Immunophenotyping of (**A**) blood cell population and (**B**) Transporter expression of Glut1. Orange PLWH without MetS and blue PLWH with MetS. Single asterisks indicate statistically significant differences $p < 0.001$ and FDR < 0.1 and double asterisk $p < 0.001$ and FDR < 0.05.
