# Supplementary material for: The central role of the glutamate metabolism in long-term antiretroviral treated HIV-infected individuals with metabolic syndrome
Source: Aging (Albany NY). 2021 Oct 11;13(19):22732–51. doi: 10.18632/aging.203622 (PMC8544298; doi:10.18632/aging.203622)
Supplement: Supplementary Tables [file aging-13-203622-s002.pdf]

SUPPLEMENTARY TABLES

Supplementary Table 1. Linear regression models adjusted for BMI and treatment regimen (PI, NNRTI and INSTI) showing the association of 11 biomarkers with metabolic syndrome in PLWH.

	Estimate	Std Error	tStat	*P* value	*P* adjust
**X1.CARBOXYETHYLLEUCINE**	0.485	0.141	3.456	<0.001	0.001
**X4.CHOLESTEN.3.ONE**	0.198	0.059	3.307	0.001	0.001
**X4.HYDROXYGLUTAMATE**	1.412	0.332	4.252	<0.001	<0.001
**ALPHA.KETOGLUTARATE**	0.18	0.053	3.368	<0.001	0.001
**CAROTENE.DIOL.2.**	–0.34	0.081	–4.196	<0.001	<0.001
**GAMMA.GLUTAMYLGLUTAMATE**	0.379	0.068	5.545	<0.001	<0.001
**GLUTAMATE**	0.393	0.072	5.386	<0.001	<0.001
**GLYCERATE**	–0.169	0.047	–3.544	<0.001	<0.001
**ISOLEUCINE**	0.093	0.028	3.295	0.002	0.001
**PALMITOYL.SPHINGOSINE.PHOSPHOETHANOLAMINE.D18.1.16.0**	–0.157	0.036	–4.415	<0.001	<0.001
**PIMELOYLCARNITINE.3.METHYLADIPOYLCARNITINE.C7.DC.**	–0.396	0.134	–2.962	0.003	0.003

Supplementary Table 2. Linear regression models adjusted for BMI and treatment regimen (AZT, TDF, TAF and ABC) showing the association of 11 biomarkers with metabolic syndrome in PLWH.

	Estimate	Std Error	tStat	*P* value	*P* adjust
X1.carboxyethylleucine	0.426	0.152	2.797	0.006	0.006
X4.cholesten.3.one	0.268	0.063	4.194	<0.001	<0.001
X4.hydroxyglutamate	1.332	0.365	3.65	<0.001	<0.001
alpha.ketoglutarate	0.151	0.058	2.597	0.01	0.0101775
carotene.diol..2.	–0.339	0.089	–3.777	<0.001	<0.001
gamma.glutamylglutamate	0.318	0.072	4.427	<0.001	<0.001
glutamate	0.314	0.077	4.06	<0.001	<0.001
glycerate	–0.147	0.047	–3.154	0.002	0.003
isoleucine	0.089	0.03	2.947	0.004	0.004
palmitoyl.sphingosine.phosphoethanolamine..d18.1.16.0.	–0.152	0.038	–3.948	<0.001	<0.001
pimeloylcarnitine.3.methyladipoylcarnitine..C7.DC.	–0.449	0.145	–3.082	0.002	0.003

Supplementary Table 3. Linear regression models adjusted for xenobiotics levels showing the association of 10 biomarkers with metabolic syndrome in PLWH.

	Estimate	Std Error	tStat	*P* value	*P* adjust
1-carboxyethylleucine	0.274	0.145	1.878	0.062	0.068
4-cholesten-3-one	0.533	0.144	3.687	<0.001	<0.001
4-hydroxyglutamate	0.533	0.143	3.723	<0.001	<0.001
alpha-ketoglutarate	0.447	0.148	3.025	0.003	0.003
carotene diol (2)	–0.459	0.144	–3.191	0.002	0.003
gamma-glutamylglutamate	0.719	0.133	5.376	<0.001	<0.001
glutamate	0.743	0.134	5.511	<0.001	<0.001
glycerate	–0.036	0.088	–0.412	0.68	0.681
isoleucine	0.413	0.146	2.816	0.005	0.007
palmitoyl-sphingosinephosphoethanolamine (d18:1/16:0)	–0.669	0.141	–4.739	<0.001	<0.001
pimeloylcarnitine/3- methyladipoylcarnitine (C7-DC)	–0.469	0.149	–3.147	0.002	0.003

Supplementary Table 4. List of antibodies for flow cytometry.

CD4-BUV395-SK3	BD Biosciences	563552
CD8-APC-RPA-T8	Biolegend	301014
CD14-BV510-M5E2	Biolegend	301842
CD3-BV711-OKT3	Biolegend	317328
CD16-BV786-3G8 (RUO)	BD Biosciences	563690
GLUT1-FITC-# 202915	R&D Systems	FAB1418F
xCT-AF594	Novus Biologicals	NB300-318AF594
MCT1/SLC16A1 AF405-# 882616	R&D Systems	FAB8275V-100UG
